# Protocol for endothelial cell-derived extracellular vesicle isolation and characterization

**DOI:** 10.1016/j.xpro.2026.104887

**Published:** 2026-10-09

**Authors:** Charlotte Hooper, Naveed Akbar

**Affiliations:** 1 Division of Cardiovascular Medicine, Radcliffe Department of Medicine, University of Oxford, OX3 9DU Oxford, UK

**Keywords:** Cell Biology, Molecular Biology, Biotechnology and bioengineering

## Abstract

Endothelial cells regulate intercellular and inter-organ communication through extracellular vesicles (EVs), which have diagnostic and therapeutic potential. We describe single-particle interferometric reflectance imaging directly from culture supernatants, followed by ultracentrifugation isolation of EVs. We determine EV size and concentration by nanoparticle tracking analysis and characterize EVs by western blotting and transmission electron microscopy. This protocol can be applied to other cell systems of EV biology.

For details on the execution of this protocol, please refer to Timms et al.^1^

## Highlights

• Direct characterization of endothelial EVs in conditioned medium
• Stepwise isolation of endothelial EVs by ultracentrifugation
• Multimodal EV characterization by NTA, western blotting, TEM, and NanoView

## Abstract (editor highlights)

Publisher’s note: Undertaking any experimental protocol requires adherence to local institutional guidelines for laboratory safety and ethics.

## Graphical abstract

**Figure:**
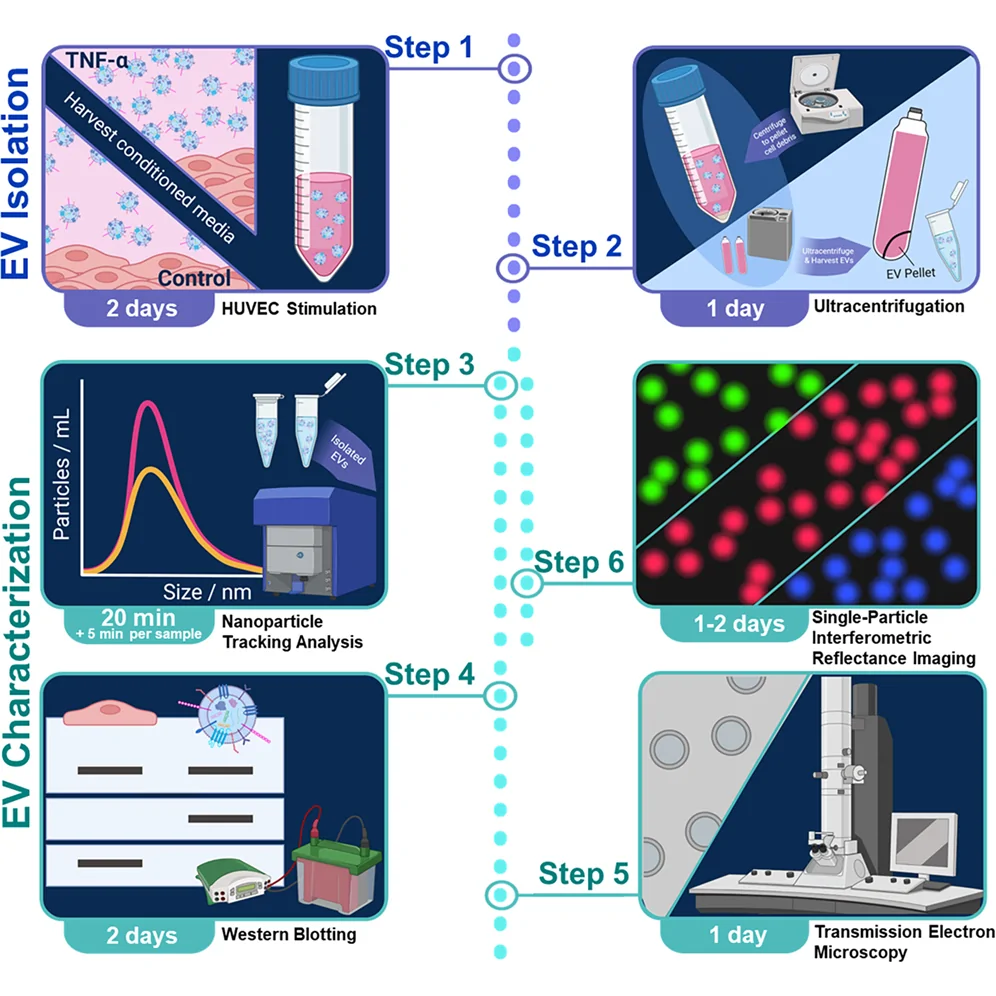

## Before you begin

Inter-organ communication is mediated by circulating signals, including nucleic acids, proteins, and lipids, which are transported in part by extracellular vesicles (EVs). Studies of isolated EVs have provided insight into their roles in health and disease and have advanced understanding of EV-mediated cellular communication.

Previous work from our group has demonstrated an increase in vascular cell adhesion molecule-1 (VCAM-1)-expressing EVs in the peripheral blood of patients and mice following myocardial infarction (MI), as well as from activated endothelial cells in culture. These EVs interact with immune cells in the spleen, leading to transcriptional activation and mobilization of these immune cells into the peripheral blood. The mobilized immune cells are subsequently recruited to sites of myocardial injury, where they may modulate inflammatory responses and contribute to tissue injury and repair.^2^˒^3^

In addition, EV cargo reflects disease states. For example, miRNA-320b, miRNA-126-3p and miRNA-126-5p are enriched in plasma EVs from patients with MI and are increased in EVs released from endothelial cells following tumor necrosis factor (TNF)-α stimulation.^1^^,^^2^^,^^3^ These EVs enhance monocyte adhesion, promote gene expression, and modulate cytokine production in peripheral blood monocyte-derived macrophages.^1^

These applications rely on consistent EV isolation and standardized downstream characterization. The protocol described here provides a workflow for the generation, isolation, and characterization of endothelial cell–derived EVs from cell culture systems.

### Innovation

Here, we describe a complete approach for the integration of direct single-particle characterization of EVs in conditioned medium from primary endothelial cells, prior to isolation by ultracentrifugation, followed by standardized approaches for orthogonal characterization by nanoparticle tracking analysis (NTA), Western blotting, and transmission electron microscopy (TEM). This protocol was optimized using human umbilical vein endothelial cells (HUVECs), but can be applied to other sources, including culture supernatants from additional cell lines and patient plasma. Similar approaches have been used to isolate EVs from mouse endothelial cell lines (sEND.1),^3^ as well as from adipocytes^4^ and vascular smooth muscle cells.^5^

A distinguishing feature of this protocol is that EVs can be characterized directly in conditioned medium before isolation, enabling comparison with the subsequently isolated EV population using complementary analytical approaches and reducing reliance on any single method for EV characterization.

Stimulation of endothelial cells with TNF-α enhances EV release and yields vesicles enriched in vascular cell adhesion molecule-1 (VCAM-1).^1^ For isolation, cell culture supernatants are first subjected to low-speed centrifugation to remove cellular debris, followed by ultracentrifugation to sediment EVs. Vesicle size and concentration are determined using single-particle interferometric reflectance imaging and Nanoparticle Tracking Analysis. Further characterization is performed by Western blotting to assess EV and cellular markers, and by TEM to confirm vesicle morphology.

The workflow presented brings together existing EV isolation and analysis methodology into a concise protocol that facilitates the concomitant characterization and evaluation of EVs in accordance to current Minimal Information for Studies of Extracellular Vesicles (MISEV) guidelines,^6^ for which all studies and reporting of EV should adhere to.

### HUVEC culture

**Timing: Days to weeks (dependent on the rate of cell growth and the final number of T175 flasks required)**

Human umbilical vein endothelial cells (HUVECs) are expanded to generate enough to seed T175 flasks at 4 × 10^6^ cells per flask.

1. Culture HUVECs in 15–20 mL EGM-2 medium supplemented with BulletKit in a humidified incubator at 37°C with 5% CO_2_.
2. Passage HUVECs at 80%–90% confluency using a 1:3 split ratio.
  a. Remove culture medium.
  b. Wash cells with 10 mL phosphate-buffered saline (PBS) at RT.
    **CRITICAL:** Allow PBS to sit on the cells for 3 min at RT, then discard PBS. This is to remove soluble proteins, which enhances Trypsin efficiency in these primary endothelial cells.
  c. Add 5 mL 0.05% trypsin-EDTA and incubate at 37°C with 5% CO_2_ for 5 min.
    i. Incubate and monitor cell detachment under a microscope.
    ii. Once cells begin to detach, add 10 mL EGM-2 medium supplemented with BulletKit and gently resuspend.
      ***Note:*** The 0.05% trypsin-EDTA is prepared by diluting 0.5% trypsin-EDTA in 1X PBS. The final working solution of 0.05% trypsin-EDTA is stored at −20°C in aliquots.
  d. Centrifuge the cell suspension at 500 *× g* for 5 min at between 20°C and 25°C (68°F to 77°F).
  e. Remove and discard the supernatant.
  f. Resuspend the cell pellet in fresh EGM-2 medium supplemented with BulletKit and re-plate at the desired density.
    ***Note:*** Maintain cells below full confluency to preserve endothelial phenotype.
    ***Note:*** Use HUVECs up to absolute passage number 6 (they arrive as passage 2 from the supplier).

### Generation of extracellular vesicle-depleted fetal bovine serum

**Timing: 2 days**

Fetal bovine serum (FBS) contains EVs that can mask in vitro culture derived EV samples and confound analysis of cell-derived vesicles. To minimize this, depleting EVs from FBS prior to its use in cell culture and subsequent collection of conditioned media for vesicle isolation.

***Note:*** Commercial EV–depleted FBS (e.g., ThermoFisher Scientific UK Ltd, A2720803) can be used as an alternative.

**CRITICAL:** Apart from steps required to be undertaken in a fume hood, all other steps are performed in a biosafety cabinet to maintain sterility.

3. Transfer FBS into 13 mL Beckman Coulter ultracentrifugation tubes.
  a. Using a 10 mL syringe fitted with a 16 G x 1 ½” needle, carefully fill ultracentrifugation tubes with FBS (13 mL per tube; Figure 1A).

**Figure 1:**
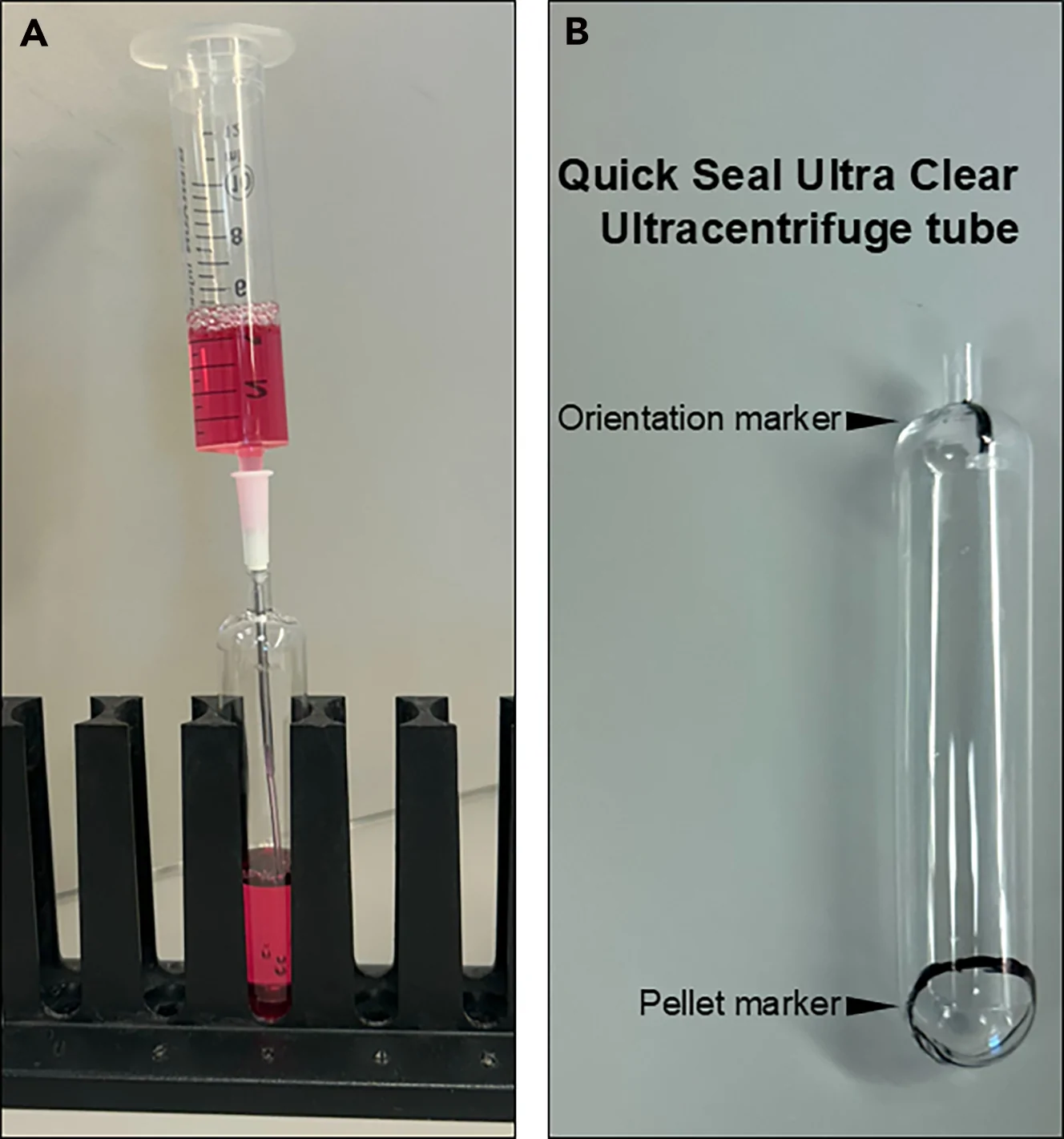
Handling of Quick Seal Ultra Clear Ultracentrifugation tubes for isolation of EVs via ultracentrifugation (A) Quick Seal Ultra Clear Ultracentrifugation tubes are filled by using a 10 mL syringe fitted with a 16 G x 1 ½” needle. (B) Quick Seal Ultra Clear Ultracentrifugation tubes are labelled with markers for pellet location at the bottom and orientation at the top prior to ultracentrifugation.
  b. Seal the tubes using a soldering iron in a fume hood.
4. Perform ultracentrifugation to deplete FBS-EVs.
  a. Ultracentrifuge at 120,000 *× g* at 4°C for 18 h (rotor type: MLA-55, k-factor: 126.5, acceleration/deceleration: set to maximum).
    **CRITICAL:** Ensure tubes are precisely balanced before centrifugation.
  b. Following centrifugation, disinfect the exterior of the tubes with 70% ethanol and transfer to a biosafety cabinet. Carefully pierce the top of each tube using a 25-gauge needle attached to a 10 mL syringe.
    **CRITICAL:** Take care when piercing tubes; keep hands clear of the needle.
  c. Withdraw approximately 5 mL of supernatant to create an air gap at the top of the tube.
  d. Carefully cut the top of the tube (∼2 cm) using scissors.
  e. Collect the remaining supernatant without disturbing the pellet.
  f. Aliquot EV–depleted FBS into 10 mL volumes and store at −20°C.
    **CRITICAL:** Avoid repeated freeze-thawing of EV-depleted FBS.
    **CRITICAL:** Avoid disturbing the pellet during supernatant collection by leaving approximately 3 mL of FBS in the bottom of the ultracentrifugation tube. Discard this remaining volume.
    **CRITICAL:** To ensure consistency between aliquots, combine equal volumes of the first 5 mL of recovered supernatant (upper portion) and the subsequent 5 mL (lower portion). Carefully collect the lower portion without approaching or disturbing the pellet. Leave approximately 3 mL above the pellet and discard this remaining volume.

## Key resources table

**Table:** 

REAGENT or RESOURCE	SOURCE	IDENTIFIER
**Antibodies**		

All listed primary antibodies are displayed with immunogen host. All listed primary antibodies target human.		
Mouse Anti-CD9 (Tetraspanin) Dilution 1 in 1000	Millipore	Cat#CBL162
Rabbit Anti-GM130 (Golgi marker) Dilution 1 in 1000	Abcam	Clone:EP892Y Cat#ab52649
Rabbit Anti-Syntenin-1 (Luminal marker) Dilution 1 in 1000	Abcam	Clone: EPR8102 Cat#ab133267
Mouse Anti-TSG101 (Luminal marker) Dilution 1 in 2000	Abcam	Clone:4A10 Cat#ab83
Rabbit Anti-VCAM-1 (Endothelial cell activation marker) Dilution 1 in 1000	Abcam	Clone: EPR5047 Cat#ab134047
Anti-Mouse IgG (H+L), HRP conjugate Dilution 1 in 20000	Promega	W402b
Anti-Rabbit IgG (H+L), HRP conjugate Dilution 1 in 50000	Promega	W401b

**Chemicals, peptides, and recombinant proteins**		

NanoStandards™ 0,100 μm Cert mean diameter: 0,096 μm Exp. Uncertainty: 0,010 μm Size CV: 5,8 % Solids: 1,0 %	Applied MicroSpheres	Cat#75009-03 Lot:WS0024.2212
Recombinant Human tumour necrosis factor-α	Biotechne	Cat#210-TA-020/CF
Amersham™ ECL™ Rainbow™ Marker - Full range	VWR International Ltd	Cat#RPN800E
EGM®-2 media supplemented with BulletKit	Lonza	Cat#CC-3162
NuPAGE MOPS SDS Running Buffer (20X), 500 mL	ThermoFisher Scientific UK Ltd	Cat#NP0001
Supersignal West Dura Extended Duration Substrate	ThermoFisher Scientific UK Ltd	Cat#34076
RIPA Buffer (10X)	Cell Signalling	Cat#9806
Fetal Bovine Serum, value, heat inactivated, One Shot™ format, 10 x 50 mL	ThermoFisher Scientific UK Ltd	Cat#A5209502
Trypsin-EDTA (0.5%), no phenol red	ThermoFisher Scientific UK Ltd	Cat#15400054
NuPAGE 4 to 12%, Bis-Tris, 1.0 mm, Mini Protein Gel, 10-well, 10 gels (1 box)	Life Technologies Ltd	Cat#NP0321BOX
cOmplete™, Mini, EDTA-free Protease Inhibitor Cocktail	Merck Life Science UK	Cat#4693159001
PhosSTOP, 20 Tablets	Merck Life Science UK	Cat#4906837001
iBlot™ 2 Transfer Stacks, nitrocellulose, regular size, 10 stacks	ThermoFisher Scientific UK Ltd	Cat#IB23001
PBS, pH 7.4, 10 x 500 mL	ThermoFisher Scientific UK Ltd	Cat#10010056
NuPAGE LDS sample buffer (4x)	ThermoFisher Scientific UK Ltd	Cat#NP0007
Uranyl acetate∗ ∗Please contact The Sir William Dunn School of Pathology, Electron Microscopy (EM) Facility for details on the full SOP. A suitable alternative is listed below:	Unknown	Unknown
UA-Zero non-radioactive EM Stain	Agar Scientific	Cat#R1260A

**Experimental models: Cell lines**		

Human Umbilical Vein Endothelial Cells, Pooled, in EGM®-2 Media	Lonza	Cat#C2519A

**Other**		

Beckman Coulter, Optima MAX – XP Ultracentrifuge	Beckman Coulter	N/A
Quick Seal Ultra Clear Ultracentrifuge Tubes	Beckman Coulter	Cat#344322
Syringe 10 mL Injekt Luer Solo	Merck Life Science UK	Cat#4606108V
Syringe 1 mL Luer	BD Plastipak	Cat#303172
Needle 25G x 5/8″	BD Microlance	Cat#300600
Needle 16G x 1 1/2″	BD Microlance	Cat#300637
ZetaView	Particle Metrix	N/A
Bio-Rad ChemiDoc MP Imaging system	Bio-Rad	N/A
Leica EM ACE 200	Leica Biosystems	N/A
120kV Tecnai 12 TEM Transmission Electron Microscope	ThermoFisher Scientific UK Ltd	N/A
OneView digital camera	Gatan	N/A
Grids (300 mesh Cu carbon film)	TAAB	Cat#C267
Whatman® qualitative filter paper, Grade 1	Merck Life Science UK	Cat#WHA1001090
Leprechaun Exosome Human Tetraspanin Kit	Unchained Labs	Cat#251-1044

**CRITICAL:** Uranyl acetate is hazardous and should be handled and disposed of in accordance with institutional safety procedures and applicable local regulations.

## Materials and equipment

**Table:** HUVEC Culture Medium (EGM-2 media supplemented with BulletKit)

Reagent	Final concentration	Amount
EGM®-2 Basal Medium	N/A	500 mL
EGM®-2 SingleQuots® Supplements: FBS	2 %	10 mL
EGM®-2 SingleQuots® Supplements: Hydrocortisone	N/A	0.20 mL
EGM®-2 SingleQuots® Supplements: hFGF-B	N/A	2 mL
EGM®-2 SingleQuots® Supplements: VEGF	N/A	0.5 mL
EGM®-2 SingleQuots® Supplements: R3-IGF-1	N/A	0.5 mL
EGM®-2 SingleQuots® Supplements: Ascorbic Acid	N/A	0.5 mL
EGM®-2 SingleQuots® Supplements: hEGF	N/A	0.5 mL
EGM®-2 SingleQuots® Supplements: GA-1000	N/A	0.5 mL
EGM®-2 SingleQuots® Supplements: Heparin	N/A	0.5 mL
**Total**	**N/A**	**515.2 mL**

Storage: Store at 4°C. Use within 1-2 weeks.

**Table:** HUVEC EV-depleted Culture Medium (EV-depleted media)

Reagent	Final concentration	Amount
EGM®-2 Basal Medium	N/A	500 mL
EV-depleted FBS	2 %	10 mL
EGM®-2 SingleQuots® Supplements: Hydrocortisone	N/A	0.20 mL
EGM®-2 SingleQuots® Supplements: hFGF-B	N/A	2 mL
EGM®-2 SingleQuots® Supplements: VEGF	N/A	0.5 mL
EGM®-2 SingleQuots® Supplements: R3-IGF-1	N/A	0.5 mL
EGM®-2 SingleQuots® Supplements: Ascorbic Acid	N/A	0.5 mL
EGM®-2 SingleQuots® Supplements: hEGF	N/A	0.5 mL
EGM®-2 SingleQuots® Supplements: GA-1000	N/A	0.5 mL
EGM®-2 SingleQuots® Supplements: Heparin	N/A	0.5 mL
**Total**	**N/A**	**515.2 mL**

Storage: Store at 4°C. Use within 1-2 weeks.

**Table:** 1X Protein lysis buffer

Reagent	Final concentration	Amount
10X RIPA	N/A	1 mL
cOmplete™ Mini EDTA-free Protease Inhibitor Cocktail	N/A	1 tablet
PhosSTOP	N/A	1 tablet
ddH_2_O	N/A	9 mL
**Total**	**N/A**	**10 mL**

Storage: Store at 4°C. Use within 1 week.

**Table:** 4X Protein lysis buffer

Reagent	Final concentration	Amount
10X RIPA	N/A	4 mL
cOmplete™ Mini EDTA-free Protease Inhibitor Cocktail	N/A	1 tablet
PhosSTOP	N/A	1 tablet
ddH_2_O	N/A	6 mL
**Total**	**N/A**	**10 mL**

Storage: Store at 4°C. Use within 1 week.

ZetaView settings^1^^,^^3^^,^^7^

• 11 positions, 2 cycles, medium video resolution.
• Pre-acquisition settings: sensitivity 80, frame 30, shutter speed 100.
• Post-acquisition settings: min brightness 25, max size 1000, min size 5, trace length 15, nm/class 30, classes/decade64.
• The ZetaView Software standard operating procedure (SOP) should be set to measure particle number and size at a shutter speed of 100 and camera sensitivity of 80, 11 camera positions for 2 cycles.

TEM settings^1^^,^^3^^,^^7^

• 120kV Tecnai 12 TEM equipped with a OneView digital camera (Gatan).

## Step-by-step method details

### Isolation of extracellular vesicles by ultracentrifugation

**Timing: 3 days**

This section describes stimulation of endothelial cells, collection of conditioned media, and isolation of EVs by differential centrifugation and ultracentrifugation.

1. Preparation of HUVECs in EV-depleted media and treatment with TNF-α:
  a. Incubate HUVECs seeded at 4 x 10^6^ cells per T175 flask overnight in complete media.
  b. Remove media from HUVECs and wash in phosphate buffered saline (PBS) (allow PBS to sit on the cells for 3 min, then discard PBS).
  c. Add 20 ml EV-depleted media ± 10 ng/mL TNF-α (or PBS vehicle) to each T175 cm^2^ flask.
    i. Incubate for 16-18 h.
2. Collection of conditioned media and cell counts:
  a. In tissue culture hood, pour cell culture supernatant from each T175 flask into a 50 mL Falcon tube.
  b. Centrifuge at 1000 *x g* at 4°C for 10 min to remove cellular debris.
  c. Pour the supernatant to a new 50 mL Falcon tube without disturbing the cell pellet.
    **CRITICAL:** Take caution to not disrupt the debris pellet.
    ***Optional:*** Retain aliquots for downstream assays (e.g., ELISA or single-particle analysis).
    In parallel, collect cells for counting.
    i. Wash cells in each flask with 10 mL PBS.
    ii. Remove PBS and replace with 0.05% Trypsin and incubate at 37°C for 10 min. Gently tap flask to dislodge the cells from the flask.
    iii. Add 1 mL FBS and 9 mL of PBS into each flask and wash the cells from the surface.
    iv. Transfer cell suspension to a 50 mL Falcon tube.
    v. Centrifuge at 500 *x g* for 5 min.
    vi. Remove supernatant and discard.
    vii. Resuspend cell pellet in 1 mL PBS and count Total, Live and Dead cells.
      ***Note:*** The 0.05% trypsin-EDTA is prepared by diluting 0.5% trypsin-EDTA in 1X PBS. The final working solution of 0.05% trypsin-EDTA is stored at −20°C in aliquots.
      ***Optional:*** Store cell pellets at −80°C if required for downstream analysis.
3. Ultracentrifugation for EV isolation:
  a. Mark a 13 mL Beckman Coulter ultracentrifugation tube with the expected EV pellet location and top for orientation (Figure 1B).
  b. Using a 10 mL syringe fitted with a 16 G x 1 ½” needle, fill one ultracentrifugation tube with the cleared supernatant from one T175 flask (Figure 1A).
    ***Note:*** Only 13 mL of the cleared supernatant from each tube is used from each T175 flask to fill one 13 mL Beckman Coulter ultracentrifugation tube. The remaining cleared supernatant is stored for downstream assays, as detailed in the “Optional” point after Step 2c.
  c. In a fume hood, seal the ultracentrifugation tube using a soldering iron.
4. Ultracentrifuge at 120,000 *× g* at 4°C for 2 h (rotor type: MLA-55, k-factor: 126.5, acceleration/deceleration: set to maximum).

**CRITICAL:** Ensure tubes are precisely balanced prior to centrifugation.

**CRITICAL:** Handle tube carefully after centrifugation to avoid disturbing the pellet.

5. Carefully remove supernatant.
  a. After centrifugation, pierce the top of the ultracentrifugation tube with a 25 G needle attached to a 10 mL syringe.
    **CRITICAL:** Care should be taken using needle to pierce tube. Keep fingers away.
  b. Remove around 1 mL of the supernatant from the top of the tube, and keep this as a sample of EV-depleted supernatant (store at −80°C).
  c. Remove a further 4-5 mL of the supernatant and discard.
  d. Carefully cut the top off the ultracentrifugation tube (around 2 cm) using scissors.
  e. In one swift motion pour off and discard the remainder of the supernatant, and allow the ultracentrifugation tube to hang upside down for 1 min.
    **CRITICAL:** Take caution during these steps, so as not to disrupt the pellet.
  f. Dab the opening of the ultracentrifugation tube with paper towel to remove excess supernatant.
  g. Resuspend the EV pellet in 100 μL PBS by scraping the pellet region with a pipette tip.
  h. Vortex briefly and gently to ensure complete resuspension.
    **CRITICAL:** The pellet may not be visible; therefore, it is important to scrape circled area and vortex.
    ***Note:*** Refer to troubleshooting problems 1 and 2.
  i. Transfer isolated EVs to micro-centrifuge tubes.
    **Pause point:** Isolated EVs can be stored on ice short-term (1-2 days) or for long-term storage, isolated EVs can be stored at −80°C (we have not tested the duration of long-term storage on isolated EVs).
6. Washing and pooling EVs.
  a. Mark a 13 mL Beckman Coulter ultracentrifugation tube with the expected EV pellet location and top for orientation (Figure 1B).
  b. Using a 10 mL syringe fitted with a 16 G x 1 ½” needle, fill one ultracentrifugation tube per condition (Control and TNF-α stimulated) with 10 mL PBS (Figure 1A).
  c. Combine the isolated EVs from the same condition, and add to the ultracentrifugation tube containing PBS.
  d. Seal the ultracentrifugation tube using a soldering iron.
  e. Ultracentrifuge at 120,000 *× g* at 4°C for 1 h (rotor type: MLA-55, k-factor: 126.5, acceleration/deceleration: set to maximum).
    **CRITICAL:** Ensure tubes are balanced prior to centrifugation.
    **CRITICAL:** Take caution during removal of tubes from the ultracentrifuge, so as not to disrupt the pellet.
  f. After centrifugation, pierce the top of the ultracentrifugation tube with a 25 G needle attached to a 10 mL syringe.
    **CRITICAL:** Care should be taken using needle to pierce tube. Keep fingers away.
  g. Remove around 5 mL of the supernatant from the top of the tube and discard.
  h. Carefully cut the top off the ultracentrifugation tube (around 2 cm) using scissors.
  i. In one swift motion pour off and discard the remainder of the supernatant, and allow the ultracentrifugation tube to drain upside down for 1 min.
  j. Dab the opening of the ultracentrifugation tube with paper towel to remove excess media.
    **CRITICAL:** Take caution during these steps, so as not to disrupt the pellet.
  k. Resuspend the EV pellet in 100 μL PBS.
    i. Vortex briefly to ensure pellet is fully resuspended.
    ii. Incubate samples on ice for 1 h before proceeding to NTA analysis (treat control and TNF-α the same).
      **CRITICAL:** Ensure complete resuspension of the pellet. The pellet may not be visible; therefore, it is important to scrape circled area with a pipette tip and vortex.
      ***Note:*** Refer to troubleshooting problems 1 and 2.
      ***Optional:*** If EVs are to be used for Western blotting, then the pellet can be directly resuspended in protein lysis buffer (refer to Steps 9-11 for Western blotting protocol).
      **CRITICAL:** Avoid repeated freeze–thaw cycles; use freshly isolated vesicles where required, for example for functional analysis studies *in vitro* and *in vivo*.
      ***Note:*** Refer to troubleshooting problems 1, 2, and 5.

### Nanoparticle tracking analysis to determine isolated EV concentration and size

**Timing: 20 min (Set-up and shutdown) + 5 min (*per* sample)**

Determine the size and concentration of isolated EVs.

**CRITICAL:** Before proceeding to downstream analysis protocols, particles need to be characterized as per MISEV2023 guidelines to confirm isolation of EVs. Refer to Welsh et al 2024 JEV^6^ for up-to-date guidance.

7. ZetaView set-up:
  a. Switch on theZetaView and open the software.
    ***Note:*** Ensure the ZetaView waste bottle is empty, and stock bottles of dH_2_O and PBS are full prior to starting.
    **CRITICAL:** Ensure that samples to be loaded into the ZetaView are not protein-rich, as this can clog the sample chamber and accompanying tubes. Protein-rich samples are not an issue for samples isolated via the ultracentrifugation method detailed here, however may be an issue if using other isolation techniques, such as precipitation, size exclusion chromatography and high performance liquid chromatography, for which fractions that are rich in soluble proteins should not be used.
  b. When prompted, fill a 10 mL syringe from the falcon tube of dH_2_O and inject 5 mL into the sample chamber, to fill the chamber.
    i. A cell check will be performed to measure cell quality. The readout should be very good to excellent.
      **CRITICAL:** Ensure that no air bubbles are introduced into the system throughout the protocol.
  c. Prepare 1 in 1000 stock dilution of the NanoStandards™ beads by adding 1 μL of beads to 1 mL dH_2_O.
    ***Note:*** The stock dilution of beads can be stored at 4°C for up to 1 week.
  d. Prepare the working dilution of the NanoStandards™ beads by adding 8 μL of the stock dilution of beads to 1 mL dH_2_O.
  e. When prompted, inject the working dilution of the NanoStandards™ beads into the sample chamber using a 1 mL syringe, to calibrate the ZetaView.
    ***Note:*** Auto-alignment of the camera will be performed, and voltage will be checked.
  f. Prime the sample chamber with PBS.
  g. In the software “Pump/temp” tab, click on “pump”, followed by “run” for pump 2 (confirm that on your machine set-up that pump 2 is connected to PBS).
  h. When prompted, inject 5 mL PBS into the sample chamber using a 10 mL syringe, to wash the chamber.
    **CRITICAL:** The particle count should be less than 5.^2^^,^^4^ If the particle count is too high, then repeat the wash step until it is below 5.
  i. Prepare 1 mL of sample at a 1 in 1000 dilution in PBS.
  j. Inject 1 mL of sample into the sample chamber using a 1 mL syringe.
    ***Note:*** The dilution may need to be changed, depending on sample concentrations. The ZetaView NTA has a working range between 1x10^4^ – 1x10^9^ particles per mL. Injected samples provide a live readout in the range of 50–400 particles in the field of view. The particles detected readout shows a green, amber, or red bar, depending on the number of particles in the field of view. A sample displaying a green or amber readout can be used for processing. For reference, samples containing between 0–50 particles will give an amber bar, samples containing between 50–200 particles will give a green bar, and samples containing between 200–400 particles will give an amber bar. More than 400 particles in the field of view will give a red bar, indicating dilution is necessary (perform a PBS wash step between injection of different sample dilutions).
    When a sample is injected containing particle counts above the recommended range, then a dilution into this range should be applied, for example injecting a sample with a live read out of 1200 particles in the field of view should be diluted a further 1 in 3 or 1 in 4 times. The software will give a live read out of particles in the field of view with an editable input for the dilution factor, whereby it will provide provisional calculated particles per mL prior to sample analysis. This range is required as the ZetaView tracks individual particles, when a sample contains more than 400 particles in the field of view, tracking an individual particle isn't achievable as they will cross paths with other particles during the video analysis.
    When a sample is injected containing particle counts below the recommended range, the sample should be diluted less, for example a sample containing only 20 particles in the field of view with an initial dilution of 1 in 1000 should be prepared again with a 1 in 300 dilution. If further dilutions aren’t feasible, then increasing the cycles during a measurement can increase the statistical strength of the measurement, however it is recommended that 50 particles should be the minimum particles in the field of view for a measurement.
    ***Note:*** Refer to troubleshooting problems 1 and 2.
  k. In the software, on the “measurement” tab, click “Run video acquisition”.
     When prompted, fill in details for saving data.
  l. Load the SOP (refer to materials and equipment section for full details on ZetaView settings).
  m. To begin data acquisition, click “OK”.
    **CRITICAL:** Ensure that the ZetaView and surrounding worksurface are free of vibrations during all steps.
  n. Click “Continue” to open the data PDF.
  o. Inject 5 mL PBS into the sample chamber using a 10 mL syringe, to wash the chamber.
  p. Repeat steps 7i – 7o for all samples.
8. ZetaView shut-down:
  a. When all samples have been measured, fill a 10 mL syringe with dH_2_O and inject into the sample chamber, to wash the chamber.
  b. Wash the sample chamber with dH_2_O with a further 10 mL syringe of dH_2_O.
  c. In the software “Pump/temp” tab, click on “pump”, followed by “run” for pump 1 (confirm that on your machine set-up that pump 1 is connected to dH_2_O).
  d. When complete, inject 10 mL dH_2_O into the sample chamber using a 10 mL syringe, to wash the chamber.
  e. Using an empty 10 mL syringe, inject air into the chamber several times until the waste tubing runs clear of liquid and produces bubbling.
  f. Close the ZetaView software and turn off the machine.

***Note:*** The ZetaView should undergo a deep-clean of the sample chamber, as per the manufacturer’s instructions, depending on machine usage.

### Western blotting of isolated EVs

**Timing: 2 days**

Verification of the presence of EV-associated protein markers and the absence of markers of cellular proteins via Western blotting provides an essential step in EV characterization.

9. Sodium dodecyl-sulfate polyacrylamide gel electrophoresis (SDS-PAGE).
  a. Prepare samples for gel electrophoresis in 4X NuPAGE LDS sample buffer.
    ***Note:*** Freshly isolated EVs or EVs that have been stored at −80°C can both be used for downstream analysis by Western blotting. For freshly isolated EVs, the EV pellet can be directly resuspended into 1X Protein lysis buffer and 4X NuPAGE LDS sample buffer added. Alternatively, frozen EVs that have been stored in PBS should be combined with a 4X Protein lysis buffer and 4X NuPAGE LDS sample buffer.
    ***Note:*** The tetraspanin proteins require running under non-reducing conditions and not boiled, therefore the antibodies used here have also been optimized to work under these conditions. If it is necessary to blot for additional antibodies, then it may be necessary to prepare an additional sample from the protein lysate with the addition of reducing agent. These samples prepared under reducing conditions should also be heated at 95°C for 10 min prior to loading onto the gel.
  b. Load samples onto a NuPAGE 4-12% gradient Bis-Tris Protein Gel
    i. Normalized loading is accounted for by loading equal volumes of each sample.
    ii. Run gel in 1X MOPS SDS Running Buffer at 150 V for 60 min.
10. Transfer gels onto nitrocellulose membrane using standard protocol.
  ***Note:*** Transfer was done using the ThermoFisher Scientific iBlot 2 Western Blot Transfer System at 20 V for 7 min.
  a. Stain membranes for total protein in Ponceau S for 1 min and imaged using ChemiDoc.
  b. Rinse off Ponceau S stain briefly in PBS-0.1% tween.
11. Block membrane for non-specific binding in 5% milk in PBS-0.1% tween for 1 h at between 20°C and 25°C (68°F to 77°F) on a rocker.
  a. Incubate membranes in primary antibodies diluted in 5% milk in PBS-0.1% tween overnight at 4°C on a rocker (refer to key resources table for antibody concentrations).
  b. Wash membranes 3X in PBS-0.1% tween for 10 min at between 20°C and 25°C (68°F to 77°F) on a rocker.
  c. Incubate membranes in horseradish peroxidase-conjugated secondary antibodies diluted in 5% milk in PBS-0.1% tween for 1 h at between 20°C and 25°C (68°F to 77°F) on a rocker (refer to key resources table for antibody concentrations).
  d. Wash membranes 3X in PBS-0.1% tween for 10 min at between 20°C and 25°C (68°F to 77°F) on a rocker.
  e. Incubate membranes with enhanced chemiluminescence (ECL) substrate for 1-5 min at between 20°C and 25°C (68°F to 77°F), by combining equal volumes of the two reagents.
  f. Membranes are imaged using standard blot imaging system.

***Note:*** The Bio-Rad ChemiDoc MP Imaging system was used. Imaging was done using Chemi Hi Resolution and exposure time was optimized for intense bands.

***Note:*** Refer to troubleshooting problem 3.

### Transmission electron microscopy of isolated EVs

**Timing: 1 day**

TEM permits the visualization of EV morphology and size, to enable their identification from other particles, in addition to providing insight into sample purity. TEM provides a level of EV characterization, which can be used in combination with NTA and Western blotting, to confirm the presence of EVs in samples.

**CRITICAL:** EVs should be isolated fresh. However, EVs can be isolated the day before and stored at 4°C overnight.

12. Glow discharge the grids (carbon side facing upwards) using a Leica EM ACE 200 for 20 s at 15 mA to promote adsorption of samples.
  a. Apply EVs to grids (300 mesh Cu carbon film) by passive adsorption for 2 min.
    i. Blot the grids with Whatman No 1 filter paper to remove residue.
      ***Note:*** The grids are not washed after EV adsorption or before staining.
  b. Stain the grids with 2% uranyl acetate in MiliQ water for 20 s.
    ***Note:*** The uranyl acetate is not required to be freshly made. Instead, 2% uranyl acetate working stocks are filtered into aliquots. Aliquots are centrifuged for 5 min at max. speed to pellet any uranyl acetate crystals just before use.
    ***Note:*** An alternative to uranyl acetate is UA-Zero non-radioactive EM Stain (details in the key resources table).
    i. Allow the grids to air dry.
  c. Acquire images using a 120kV Tecnai 12 TEM equipped with a OneView digital camera.
    ***Note:*** Refer to troubleshooting problem 4.

### Single-particle interferometric reflectance imaging of EVs

**Timing: 1–2 days**

Single-particle interferometric reflectance imaging enables direct characterization of EVs from conditioned media, including particle sizing and surface marker detection. The workflow below describes EV capture and fluorescent labelling using the NanoView Leprechaun platform.

13. Prepare the NanoView platform and reagents.
  a. Remove Luni chips from 4°C storage and allow them to equilibrate to between 20°C and 25°C (68°F to 77°F) for 15 min before opening the packaging.
    **CRITICAL:** Use opened Luni chips within 2 weeks of opening the Mylar packaging.
  b. Thaw conditioned media samples at between 20°C and 25°C (68°F to 77°F).
  c. Centrifuge samples at 2,500 × g for 5 min at between 20°C and 25°C (68°F to 77°F) to remove debris.
  d. Turn on the NanoView scanner, washer, and acquisition computer.
  e. Create folders for pre-scan and post-scan image acquisition.
14. Prepare samples for incubation on Luni chips.
  a. Centrifuge conditioned media samples twice at 2,500 × g for 15 min prior to analysis.
    ***Note:*** Conditioned media supplemented with EV-depleted foetal bovine serum can be analysed directly without prior EV isolation.
  b. Dilute samples 1:1 in 1× Incubation Solution.
  c. Mix 50 μL conditioned media with 50 μL incubation buffer.
15. Perform background scanning of Luni chips prior to sample incubation.
  a. Load Luni chip files into the acquisition software.
  b. Assign chip identifiers and perform pre-scanning using all three laser channels.

**CRITICAL:** Incorrect chip assignment may result in scan failure.

16. Incubate samples on Luni chips.
  a. Place Luni chips into the NanoView washer plate using forceps.
    **CRITICAL:** Avoid touching the center of the Luni surface to prevent damage to capture antibodies.
  b. Orient each Luni chip with the chip identifier facing the ramp.
  c. Add 50 μL of diluted sample onto each Luni chip.
    **CRITICAL:** Avoid introducing bubbles during sample loading.
  d. Seal the plate and incubate for 16 h at between 20°C and 25°C (68°F to 77°F) on a vibration-free surface.
17. Perform surface marker immunofluorescence staining.
  a. Remove the plate seal and place the plate into the NanoView washer.
  b. Select the “Exosome” protocol within the washer software.
  c. Prepare fluorescent antibody staining solution in blocking buffer.
  d. Add fluorescent antibodies against CD9, CD63, CD81, and isotype control at a 1:500 dilution (antibodies are supplied in the Leprechaun Exosome Human Tetraspanin Kit).
  e. Vortex briefly to mix.
  f. Add 250 μL antibody mixture to each well.
  g. Incubate for 60 min at between 20°C and 25°C (68°F to 77°F).

***Note:*** Alternative fluorescent antibodies can be substituted following optimization.

18. Perform cargo staining where required.
  a. Select the “Exosome Cargo” protocol.
  b. Add Solution C followed by Solution D from the Leprechaun Exosome Human Tetraspanin Kit according to the manufacturer’s instructions.
  c. Prepare antibody staining solution containing antibodies against CD9, CD63, CD81, and the isotype control.
  d. Add 250 μL staining solution to each well and incubate for 60 min.
19. Dry and scan Luni chips.
  a. Move Luni chips onto the drying ramp while submerged.
  b. Remove chips and allow them to dry completely on an absorbent surface.
  c. Load chips into the NanoView scanner and perform post-scan acquisition.
20. Analyze EV size and fluorescence data.
  a. Load pre-scan and post-scan files into the analysis software.
  b. Apply dilution correction factors during analysis.
  c. Set fluorescence thresholds individually for each chip.
    **CRITICAL:** Background fluorescence should remain below 50 particles for red and green channels and below 100 particles for the blue channel.
  d. Export total particle counts, size distributions, and fluorescence intensity data for downstream analysis.
    ***Note:*** Refer to troubleshooting problem 5.

## Expected outcomes

NTA, marker analysis, and TEM are essential components of EV characterization in accordance with MISEV2023 guidelines. NTA provides quantitative assessment of EV concentration and size distribution (Figure 2). Typical EV yields obtained from 20 mL of conditioned media collected from a T175 flask seeded with 4 × 10^6^ HUVECs range an average of 8611 ± 2730 SD (particles/total cell count) for control and 34370 ± 7328 SD (particles/total cell count) for TNF-α treated samples, depending on endothelial activation state and culture conditions. Particle size distributions are typically enriched between 50–250 nm, with modal particle sizes commonly ranging between 90–150 nm. Stimulation of HUVECs with TNF-α is expected to increase EV release compared with unstimulated controls and enrich vesicles expressing inflammatory endothelial markers including VCAM-1.

**Figure 2:**
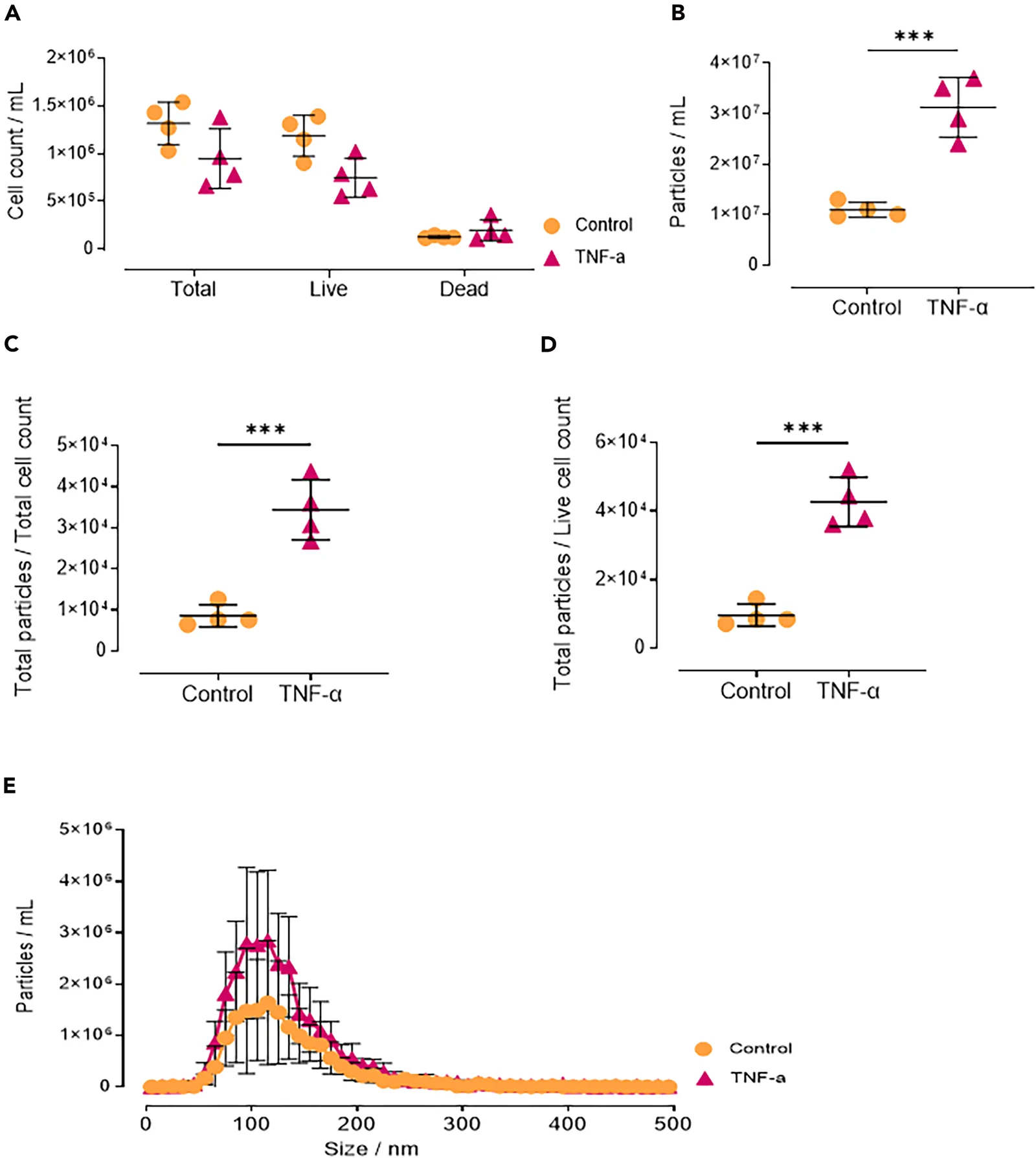
Particles isolated from HUVECs (Control and TNF-α stimulated) via ultracentrifugation (A) Total, Live, and Dead cell counts for HUVEC cells that were used to generate cell supernatants for EV isolation. (B) Total particles per mL normalized to Live cell counts, displaying increased EV concentration from TNF-α stimulated cells. (C) Total number of particles normalized to total cell counts. (D) Total number of particles normalized to live cell counts. (E) Particles per mL and size distribution. Data are expressed as mean ± SD. *N* = 4 biological replicates per group. P values were determined by Student’s unpaired *t* test. ∗∗∗ *p* < 0.001. Data taken from Timms et al.^1^

Western blotting of isolated EVs should demonstrate enrichment of EV-associated proteins including CD9, TSG101 and Syntenin-1, with minimal detection of cellular contaminants by GM130. HUVEC lysates should be included as positive controls for cellular proteins (Figure 3).

**Figure 3:**
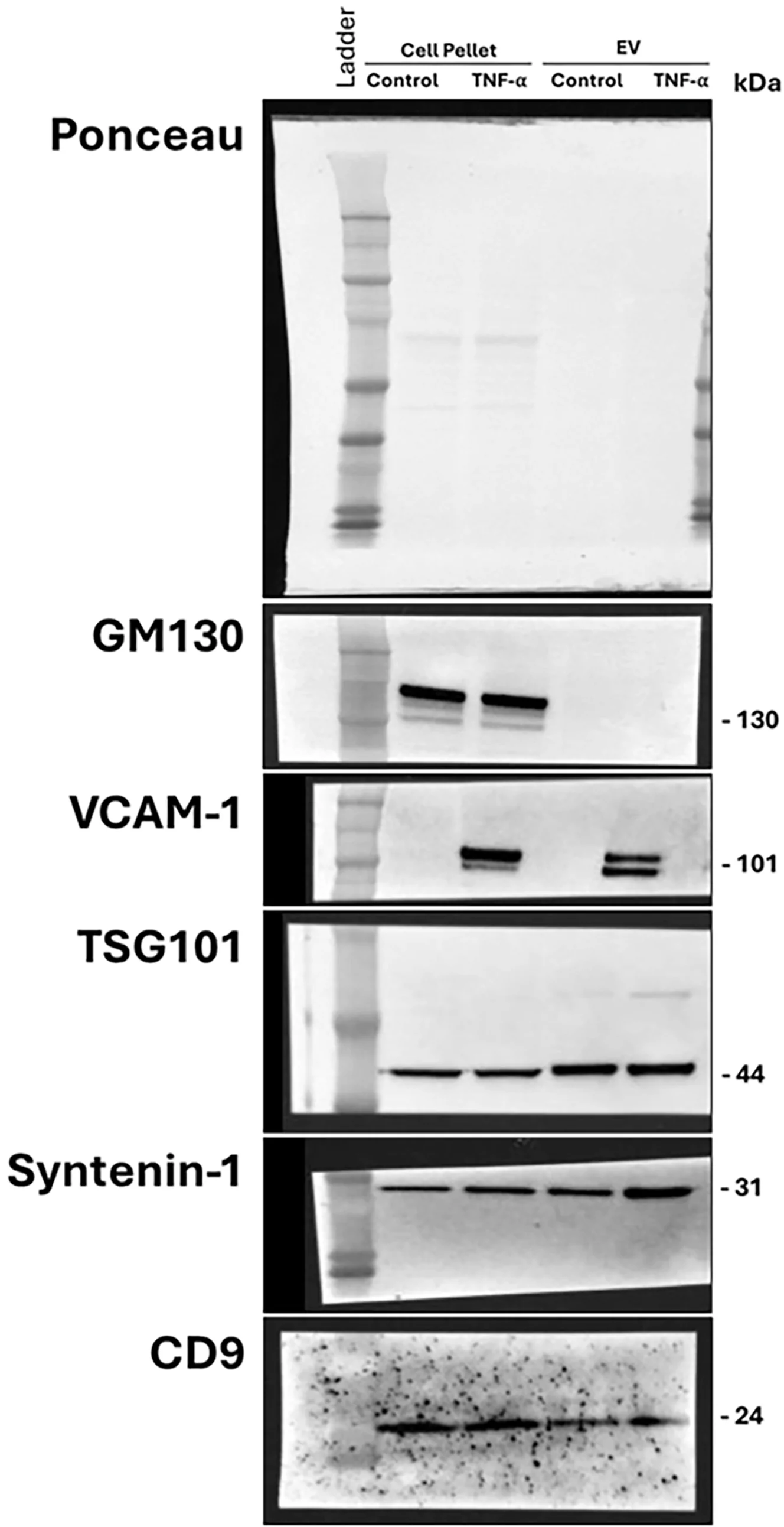
Representative uncropped Western blots of EVs isolated from HUVECs (Control and TNF-α stimulated) The EV marker proteins syntenin-1 and TSG-101 (luminal markers), and CD9 (tetraspanin) are run along with GM130 (Golgi marker) as an indication of cellular material. HUVEC cells (control and TNF-α stimulated) are run as controls. VCAM-1 expression is increased in TNF-α stimulated cells and EVs. Data taken from Timms et al.^1^

Transmission electron microscopy should demonstrate membrane-bound vesicles with characteristic rounded or cup-shaped morphology and diameters consistent with small EV populations. Representative images should demonstrate intact vesicles with minimal background debris or aggregation (Figure 4).

**Figure 4:**
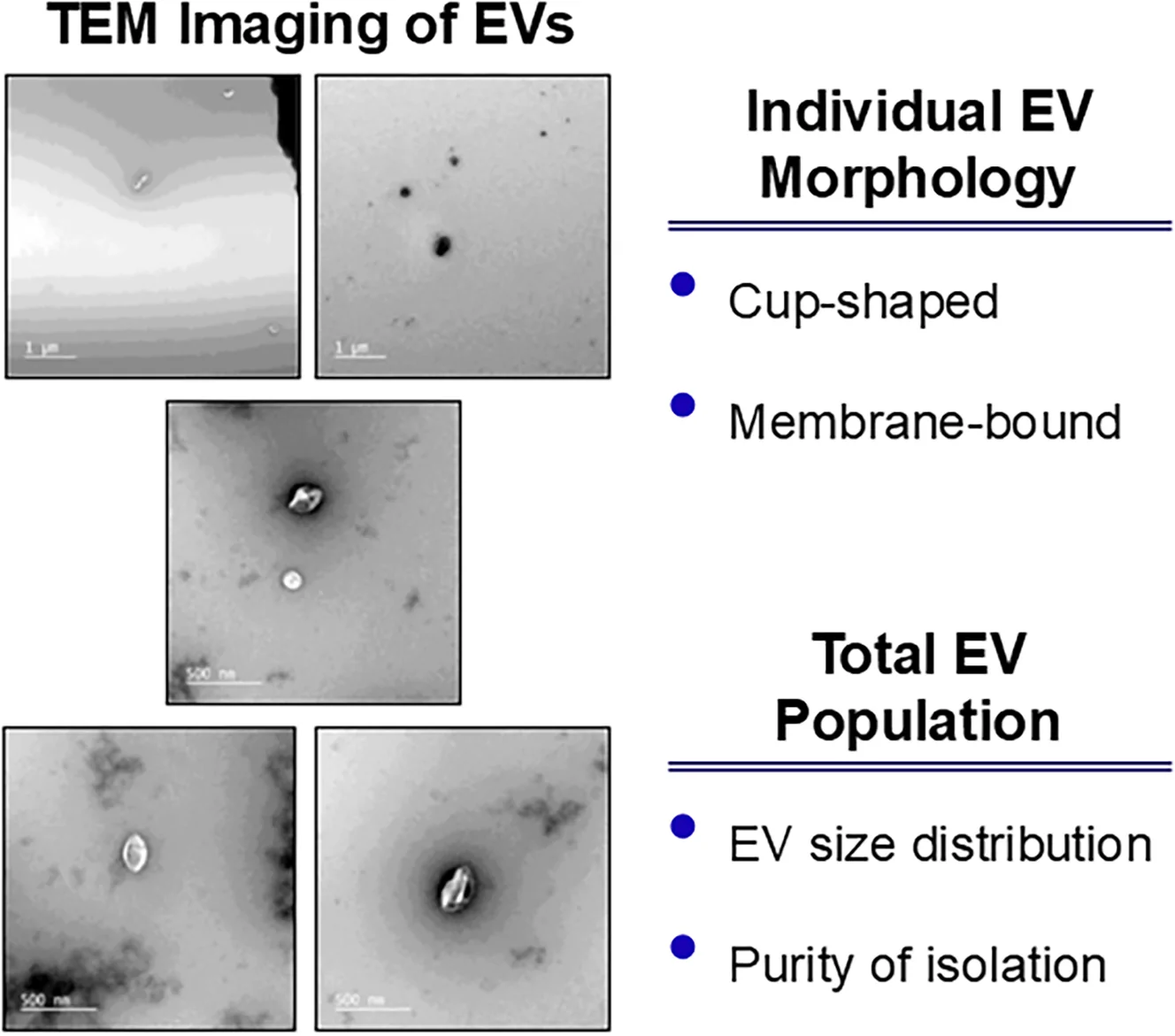
Representative transmission electron microscopy (TEM) images of EVs TEM imaging of EVs enables the visualization of individual EV morphology and visual characterization of the total EV population isolated. Parameters for EV confirmation and characterization are detailed. Scale bars 1 μm or 500 nm. Data taken from Timms et al.^1^

NanoView single-particle interferometric reflectance imaging permits direct characterization of EV surface marker expression from conditioned media prior to ultracentrifugation and is expected to demonstrate enrichment of CD9+, CD63+, and CD81+ particles following endothelial activation (Figure 5).

**Figure 5:**
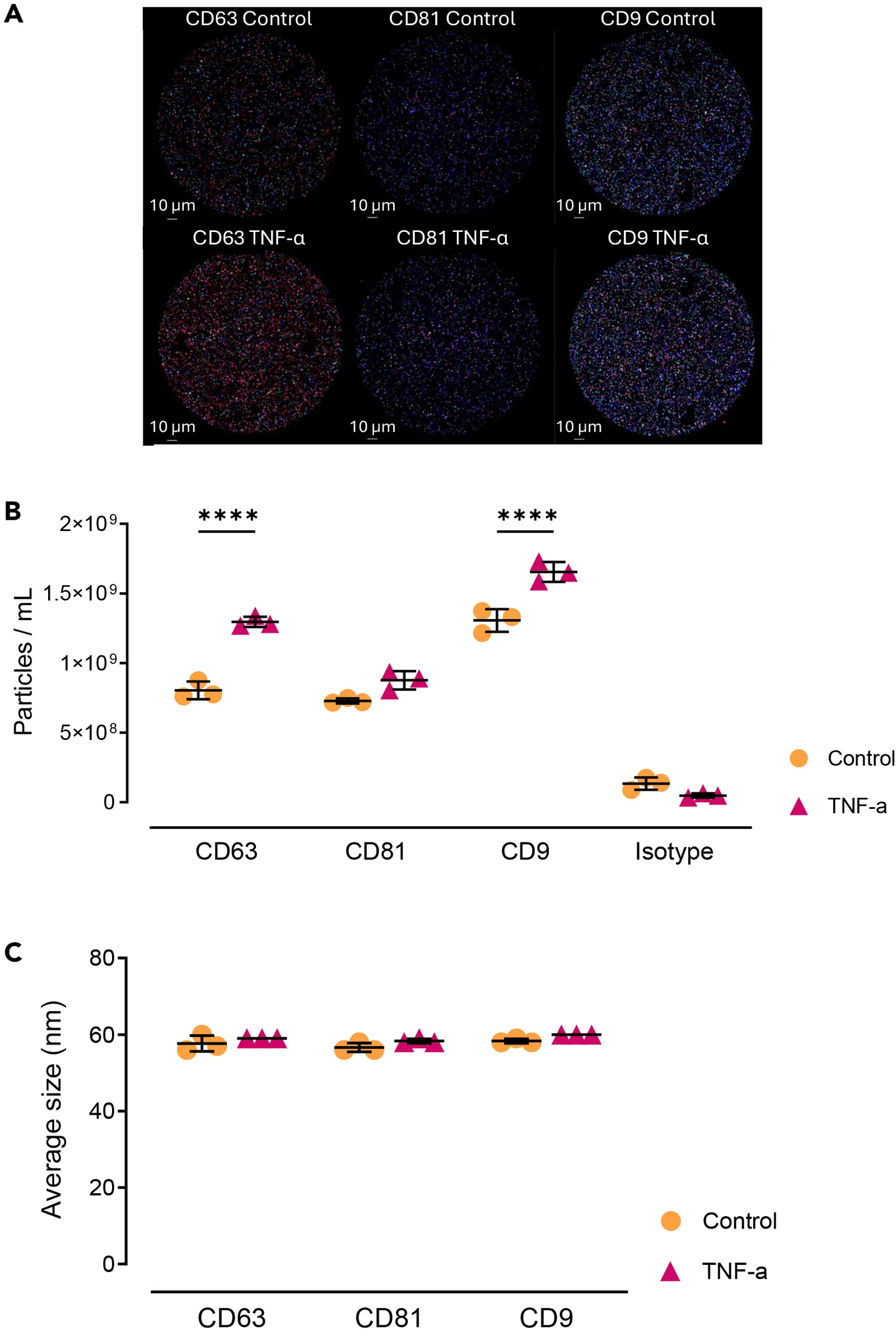
Single-particle interferometric reflectance imaging of EVs isolated from Control and TNF-α stimulated HUVECs (A) Representative images showing CD9, CD63, and CD81-positive EVs are detected for both groups. (B) Total particles per mL captured for each tetraspanin. Increased concentrations are observed with TNF-α stimulation for CD63 and CD9. (C) Average particle size captured for each tetraspanin from Control and TNF-α HUVECs. Data are expressed as mean ± SD. *N* = 4 biological replicates per group. P values were determined by two-way ANOVA with Tukey’s post hoc test. ∗∗∗∗ *p* < 0.0001. Data taken from Timms et al.^1^

## Limitations

Although this protocol has been optimized for HUVEC-derived EVs, EV yield and composition may vary substantially between endothelial cells and other biological sources. Differences in endothelial phenotype, passage number, confluency, and inflammatory activation state can influence both EV release and cargo composition.

Ultracentrifugation-based isolation may co-isolate contaminating lipoproteins, protein aggregates, or non-vesicular nanoparticles, particularly when serum depletion steps are insufficient. In addition, high centrifugal forces may promote EV aggregation or structural disruption. For applications requiring highly purified EV populations, additional purification approaches including density gradient ultracentrifugation or size exclusion chromatography may be required.

Particle sizing approaches including NTA and single-particle interferometric reflectance imaging use different detection principles and may therefore yield differences in measured particle size distributions and concentrations. Furthermore, repeated freeze-thaw cycles may compromise EV integrity and reduce functionality in downstream biological assays. For functional experiments, freshly isolated EVs are recommended where possible.

## Troubleshooting

### Problem 1

Low yield of isolated EVs (Steps 5 and 6).

### Potential solution

• Lipoprotein presence can occur, therefore, include a media-only control or culture cells in serum free conditions.
• Insufficient suspension of the EV-pellet can lead to loss of EVs during harvest, therefore ensure rigorous resuspension of the pellet.
• EVs are being measured in the incorrect range using ZetaView, so ensure correct dilution is used.
• EV production varies between cell lines; therefore, it may be necessary to scale up the number of flasks and the time of incubation for collection of cell culture supernatant. After EV isolation, EV samples from the same conditions can be combined to increase individual sample EV yield.

### Problem 2

Variable EV concentrations. Whilst it can be expected that different treatment conditions will affect EV production, and therefore final yields, it is expected that replicates within a treatment condition should be similar (Steps 5 and 6).

### Potential solution

• Insufficient suspension of the pellet can lead to loss of EVs during harvest, therefore ensure rigorous resuspension of the pellet.
• Take care not to disrupt EV-pellets.

### Problem 3

Western blotting specific to EV samples (**Steps 9-11**).

### Potential solution

• Load as many EVs as possible by increasing the volume loaded per well.
• Increase the concentration loaded by resuspending the pellet in a smaller volume of PBS, or directly into protein lysis buffer to minimize dilution of the sample.
• The tetraspanin proteins require running under non-reducing conditions, therefore the antibodies used here have also been optimized to work under these conditions. If it is necessary to blot for additional antibodies, then it may be necessary to prepare an additional sample from the protein lysate with the addition of reducing agent. These samples should also be heated at 95°C for 10 min prior to loading onto the gel.

### Problem 4

Poor TEM image quality or low EV visualization (**Step 12**).

### Potential solution

• Increase EV concentration applied to TEM grids.
• Minimize disruption during grid blotting and staining.
• Ensure uranyl acetate staining time is optimized.
• Use freshly isolated EVs where possible.
• Avoid repeated freeze-thaw cycles prior to TEM preparation.

### Problem 5

High background fluorescence during NanoView analysis (**Steps 13–20**).

### Potential solution

• Ensure conditioned media are adequately cleared by centrifugation prior to incubation on Luni chips.
• Reduce sample concentration or increase dilution factor.
• Confirm that fluorescence thresholds are applied individually for each chip during analysis.
• Ensure all buffers and incubation reagents are free from particulate contamination.
• Repeat washing steps where necessary to reduce non-specific signal.

### Problem 6

EV-poor outcomes in downstream applications.

### Potential solution

Avoid freeze/thawing of EVs. Some applications require EVs to be used fresh, in these instances the EVs can be stored overnight on ice at 4°C.

## Resource availability

### Lead contact

Further information and requests for resources and reagents should be directed to and will be fulfilled by the lead contact, Naveed Akbar (naveed.akbar@cardiov.ox.ac.uk).

### Technical contact

Technical questions on executing this protocol should be directed to and will be answered by the technical contact, Naveed Akbar (naveed.akbar@cardiov.ox.ac.uk).

### Materials availability

This study did not generate new unique reagents.

### Data and code availability

Data reported in this paper will be shared by the lead contact upon reasonable request. This paper does not report original code.

## Acknowledgments

This study was funded by British Heart Foundation Intermediate Fellowship FS/IBSRF/22/25110 (N.A.); British Heart Foundation Centre of Research Excellence, Oxford RE/13/1/30181, RE/18/3/34214, and RE/24/130024(N.A.); Oxford Biomedical Research Centre (N.A.); Nuffield Benefaction for Medicine (N.A.); Wellcome Institutional Strategic Support Fund (N.A.); Health Research Bridging Salary Scheme (N.A.); and Wellcome Accelerator Award (333500/Z/25/Z). The views expressed are those of the authors and not necessarily those of the National Health Service, the 10.13039/501100000274British Heart Foundation, or affiliated institutions. We also acknowledge the support of Facilities at the Sir William Dunn School of Pathology and Electron Microscopy Facility. BioRender was used to generate the graphical abstract.

## Author contributions

All authors contributed equally to this work. C.H. and N.A. conceived and designed the study, performed experiments, analyzed the data, and wrote the manuscript. All authors reviewed and approved the final manuscript.

## Declaration of interests

The authors declare no competing interests.
